# A Formulated TLR7/8 Agonist is a Flexible, Highly Potent and Effective Adjuvant for Pandemic Influenza Vaccines

**DOI:** 10.1038/srep46426

**Published:** 2017-04-21

**Authors:** Neal Van Hoeven, Christopher B. Fox, Brian Granger, Tara Evers, Sharvari W. Joshi, Ghislain I. Nana, Sarah C. Evans, Susan Lin, Hong Liang, Li Liang, Rie Nakajima, Philip L. Felgner, Richard A. Bowen, Nicole Marlenee, Airn Hartwig, Susan L. Baldwin, Rhea N. Coler, Mark Tomai, James Elvecrog, Steven G. Reed, Darrick Carter

**Affiliations:** 1 Infectious Disease Research Institute, 1616 Eastlake Ave E., Seattle WA 98103, USA; 2 University of California Irvine, Department of Medicine, Irvine CA 92697, USA; 3 Colorado State University Department of Biomedical Sciences, Foothills Campus, Fort Collins, CO 80523, USA; 4 3M, Inc., St. Paul, Minnesota 55121, USA

## Abstract

Since 1997, highly pathogenic avian influenza viruses of the H5N1 subtype have been transmitted from avian hosts to humans. The severity of H5N1 infection in humans, as well as the sporadic nature of H5N1 outbreaks, both geographically and temporally, make generation of an effective vaccine a global public health priority. An effective H5N1 vaccine must ultimately provide protection against viruses from diverse clades. Toll-like receptor (TLR) agonist adjuvant formulations have a demonstrated ability to broaden H5N1 vaccine responses in pre-clinical models. However, many of these agonist molecules have proven difficult to develop clinically. Here, we describe comprehensive adjuvant formulation development of the imidazoquinoline TLR-7/8 agonist 3M-052, in combination with H5N1 hemagglutinin (HA) based antigens. We find that 3M-052 in multiple formulations protects both mice and ferrets from lethal H5N1 homologous virus challenge. Furthermore, we conclusively demonstrate the ability of 3M-052 adjuvant formulations to broaden responses to H5N1 HA based antigens, and show that this broadening is functional using a heterologous lethal virus challenge in ferrets. Given the extensive clinical use of imidazoquinoline TLR agonists for other indications, these studies identify multiple adjuvant formulations which may be rapidly advanced into clinical trials in an H5N1 vaccine.

Since 1997, highly pathogenic avian influenza (HPAI) viruses of the H5N1 subtype have caused sporadic but devastating outbreaks throughout the world. Since emerging in the Guangdong province of China in 1996, the virus has spread throughout Asia into Europe, the Middle East, and Africa^1^,^2^. The sporadic transmission of H5N1 viruses into human populations from poultry or other birds has resulted in 846 clinically confirmed cases and over 449 deaths, for a case-fatality rate of 53%^3^. While H5N1 strains have yet to demonstrate the ability to transmit efficiently among people, sporadic cases of small-scale, limited human to human transmission have been reported. In addition, adaptation of the virus in a laboratory has shown that these viruses are capable of airborne transmission with a small number of identified mutations^4^,^5^,^6^. Should a transmissible H5N1 virus emerge in human populations, another pandemic would likely occur. If in addition the transmissible virus remains highly lethal, the overall public health impact of such a pandemic could be severe.

The potential for a severe H5N1 pandemic has prompted the development of several H5N1 vaccines, the majority of which focus on eliciting functional, neutralizing antibodies against the viral hemagglutinin (HA)^7^,^8^,^9^,^10^. Several of these candidates have proven to be safe in early phase clinical trials, and some have been included in pre-pandemic vaccine stockpiles to be deployed in the event an H5N1 pandemic emerges^11^. However, many of these antigens induce relatively low levels of functional neutralizing antibody when tested clinically^12^,^13^,^14^, which prompted testing of additional pandemic vaccine formulations containing adjuvants^15^. The most commonly used adjuvants in clinical stage H5N1 vaccines are alum (Alhydrogel, Adju-Phos^®^) and oil-in-water emulsions containing squalene^16^,^17^,^18^,^19^,^20^,^21^,^22^,^23^,^24^,^25^,^26^,^27^,^28^,^29^,^30^,^31^,^32^. Emulsion-adjuvanted H5N1 vaccines have been thoroughly tested clinically, and have been shown to improve vaccine potency, and to provide critical dose sparing functions.

In addition to sero-conversion against the homologous vaccine antigen, several of these studies have also examined the induction of cross-clade neutralizing antibodies to other H5N1 strains. The ability to generate functional heterologous antibodies is particularly important in the case of H5N1 vaccines insofar as the stockpiled vaccines are derived from viruses of a different clade (e.g. A/Vietnam/1203/04, Clade 1) than those which are commonly circulating.

While cross-clade neutralizing antibodies have been observed in some studies with emulsion based adjuvants^33^,^34^,^35^,^36^, the addition of other immunostimulatory agents has been explored as a means to further broaden the protective capacity of pre-pandemic H5N1 vaccines. Many of the most promising approaches include innate stimulatory molecules such as Toll-Like receptor (TLR) agonists. Agonists for TLR4, TLR7, TLR3 and other innate sensors such as RIG-I have all been tested in combination with H5N1 vaccines with promising results in pre-clinical models^37^,^38^,^39^,^40^,^41^,^42^,^43^,^44^, and have specifically been shown to stimulate broadening of antibody responses to H5N1 vaccines^45^,^46^.

The ability of imidazoquinolines to target Toll-like Receptor TLR7 and/or TLR8 to generate enhanced innate immune responses has been well documented (Reviewed in ref. 47). As synthetic small molecules, imidazoquinolines can be manufactured cost effectively and at high purity. The TLR7 ligand imiquimod is the active component in the topical cream Aldara^®^, approved for human immunotherapeutic use to treat skin cancer and genital warts^48^. However, use of injected imidazoquinolines as vaccine adjuvants have not progressed beyond clinical testing. Due to their small size, it is supposed that soluble, unformulated imidazoquinolines such as R848 rapidly diffuse from the injection site, potentially causing systemic immune activation rather than localized stimulation^49^. For this reason, strategies to “slow down” diffusion such as covalent conjugation to vaccine antigens or encapsulation in particulate formulations have shown promise in preclinical testing. Smirnov *et al*. described a chemical synthesis approach resulting in the addition of an 18-carbon chain that maintains local adjuvant activity of the imidazoquinoline but not the systemic responses evident with R848^50^. This molecule, called 3M-052 (Fig. 1), is also more amenable to incorporation in lipid-based formulations such as liposomes or emulsions. Here we describe the formulation development and influenza vaccine adjuvant activity of liposome and emulsion-based formulations of 3M-052. We find that 3M-052 based adjuvants increase the protective capacity of H5N1 antigens, promoting broadening of antibody responses, antigen dose sparing and protection in multiple pre-clinical models.

## Results

### Formulation Development of 3M-052

The structure of 3M-052 makes it amenable to formulation in a number of different lipid and oil based formulation platforms. In order to identify promising adjuvant formulations for pandemic influenza vaccines, we generated and characterized a number of different 3M-052 formulations. 1,2-dipalmitoyl-sn-glycero-3-phosphocholine (DPPC) based liposomes modified with anionic, cationic, or PEGylated phospholipids, and a neutral 1,2-dioleoyl-sn-glycero-3-phosphocholine (DOPC) based liposome were manufactured by the thin film technique followed by sonication. Liposomes were manufactured at a high 3M-052 concentration of 1 mg/ml in order to highlight any tendency for incompatibility with the various liposome compositions. The 3M-052 content is considered ‘high’ in this case since lower concentrations (e.g. 0.04 mg/mL) are adequate for the 1–2 μg doses employed in the *in vivo* studies. Although substantial variability was evident between the two separate batches for each liposome, the PEGylated liposome and the DOPC liposome demonstrated translucent appearance and the smallest particle size and polydispersity following sonication (Table 1); a translucent or semitransparent appearance is desirable for liposomes at this concentration since in our experience it is generally indicative of small particle size and low polydispersity. The PEGylated liposome was selected for further stability and *in vivo* evaluation although the DOPC liposome remains a suitable alternative. The PEGylated liposomes were negatively charged (Supplementary Table T1), most likely due to the anionic phosphate group of DPPE-PEG750. The liposomes demonstrated little or no change in particle size for at least 6 months at 5 °C (Supplementary Figure S1). In general, no loss of 3M-052 during manufacture was evident since the detected amount of 3M-052 in the manufactured product was consistent with the targeted amount added prior to manufacture.

Squalene-based oil-in-water emulsion (SE) formulations of 3M-052 were manufactured by microfluidization, generating a milky-white opaque homogeneous emulsion containing <100 nm size droplets with low polydispersity (Table 1) and good long-term particle size stability (Supplementary Figure S1) similar to previous descriptions of SE or other squalene-in-water emulsions^51^,^52^. Emulsion zeta potential values were slightly negative (Supplementary Figure S1). Interestingly, emulsions manufactured with egg PC instead of DMPC resulted in higher recovery of 3M-052 after processing (Supplementary Table T2). Moreover, adding an initial mixing step of combining 3M-052 with egg PC in chloroform further increased recovery of 3M-052 compared to sonication of the dry powder into squalene/egg PC without chloroform (Supplementary Table T2). Thus, changes in emulsion composition and processing procedure had significant effects on 3M-052 incorporation in the final formulation (Supplementary Table T2). Full recovery of 3M-052 using the optimized emulsion composition and processing was only limited at high 3M-052 concentration (e.g. 0.8 mg/mL), but this is not considered relevant since 0.04 mg/mL 3M-052 was adequate to support the 1–2 μg doses employed here.

### Formulated 3M-052 Combined with H5N1 HA Protects Mice From Lethal H5N1 Challenge Following A Single Immunization

**Figure 2:**
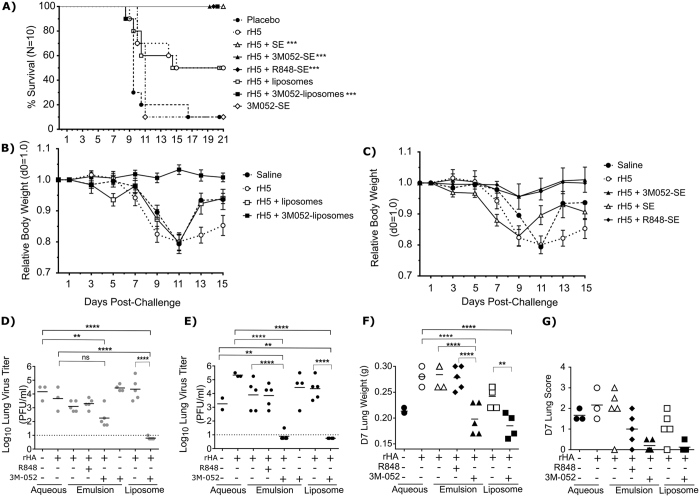
Mice immunized with 3M-052 show enhanced survival following H5N1 challenge. Mice (n = 10/group) were immunized once with rHA protein (A/VN/1203/04, Protein Sciences) in combination with adjuvants as indicated. Adjuvant dose was 1 μg R848 or 2 μg 3M-052. Twenty one days post immunization, animals were challenged with 10^6^ PFU of A/VN/1203/04 (H5N1, Clade 1) via the intra-nasal route. Animals were monitored daily for survival (**A**) and weight loss (**B**,**C**). In the case of weight loss, mean relative body weight within the group is presented, with error bars representing 1 standard deviation from the mean. 3 days (**D**) and 7 days (**E**) post-challenge, mice were euthanized, and lung virus titers determined by plaque assay. In addition, intact lungs were weighed at day 7 post-challenge as a measure of consolidation (**F**), and were scored for the appearance of gross pathology (**G**). Significance was determined by Mantel-Cox Log-Rank Test (**A**) or by One-way ANOVA, with Tukey’s correction for multiple comparisons (**D**–**G**) (**p < 0.005, ***p < 0.0005, ****p < 0.0001).

Following demonstration of the stability of 3M-052 adjuvant formulations, we investigated the ability of this TLR agonist to enhance HA specific vaccine responses. 3M-052 formulated in either PEGylated liposomes or in a squalene oil-in-water emulsion (SE) was admixed with a recombinant influenza HA H5N1 protein (rH5, A/Vietnam/1203/04 strain [VN1203], Protein Sciences Corp., Meriden CT) and used to immunize groups of C57Bl/6 mice (N = 20/group) via the intramuscular route. Twenty-one days following a single immunization, all mice were challenged intranasally with 1000 LD_50_ of VN1203. Ten animals were followed for 14 days to determine virus induced morbidity, measured by weight loss, as well as mortality. In a liposomal adjuvant formulation, 3M-052 prevented virus induced morbidity; 100% of animals receiving rH5 + 3M-052/Liposomes survived compared with 50% of animals receiving rH5 or rH5 combined with liposomes alone (Fig. 2A). Mice immunized with rH5 + 3M-052/liposomes showed no weight loss, whereas animals receiving rH5 with or without liposomes lost weight for 11 days following challenge (Fig. 2B, Supplementary Figure S2). Animals receiving emulsion based formulations showed consistent survival both with and without 3M-052 (Fig. 2A), consistent with the known efficacy of emulsions as influenza vaccine adjuvants. However, animals receiving emulsions combined with TLR 7/8 agonists showed reduced weight loss over the acute infection period compared with animals receiving rH5 + SE (Fig. 2C, Supplementary Figure S2). Mice immunized with rH5 and the non-lipidated TLR 7/8 agonist resiquimod (R848) survived as well with no evidence of weight loss.

In addition to the animals monitored for survival, 5 animals/group were euthanized at 4 and 7 days post-challenge to measure influenza induced pathology in the lung and to determine lung viral titers. At 4 days post-challenge, animals immunized with rH5 + 3M-052 adjuvant formulations had significantly reduced lung viral titers relative to non-immunized controls. Animals immunized with rH5 + 3M-052/liposomes had no detectable virus titer at this timepoint (Fig. 2D). At day 7, animals receiving 3M-052 based adjuvant formulations had minimal viral titers relative to rH5 alone. For both liposomes and SE based formulations, addition of 3M-052 resulted in significantly reduced titer relative to the same formulation without TLR (Fig. 2E). In addition to reduced viral titers, animals immunized with rH5 + 3M-052 adjuvant formulations showed significantly lower lung weight (Fig. 2F) and reduced lung pathology scores (Fig. 2G). Animals receiving rH5 and resiquimod (R848) had similar viral titers in the lung as the control mice receiving rH5 alone. Taken together, these results demonstrate the ability of 3M-052 to protect mice from clinical sequelae of lethal avian influenza challenge.

### 3M-052 Adjuvant Formulations Induce a Broadened Cross Subtype Antibody Response to H5N1 HA

**Figure 3:**
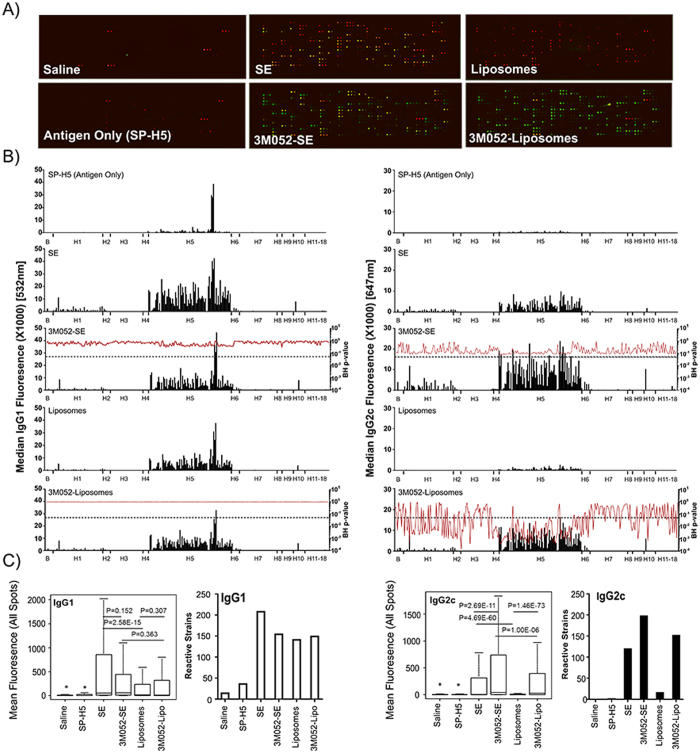
HA Subtype Binding Profile Analysis of Sera From 3M-052 Immunized Mice. Mice were immunized twice with split H5N1 vaccine (SP-H5, A/VN/1203/04, Sanofi Pasteur) 28 days apart (D0, D28) in combination with adjuvants as indicated. Adjuvant dose was 1 or 2 μg 3M-052 in SE or liposomes, respectively. Sera was collected from mice (n = 7/group) at D63, and analyzed using a high density HA array. IgG1 and IgG2 subtype antibodies were simultaneously detected using a two-color microarray scanner (**A**). Global binding profiles of serum from mice immunized with different adjuvants were determined by plotting background subtracted median fluorescence values for all HA proteins in the array. The ability of 3M-052 to significantly increase antibody titer, indicated by an increase in median fluorescence, was determined by comparison of fluorescence levels observed within a formulation (SE, Liposomes) with and without 3M-052, and calculation of P-values corrected for false discovery rate (FDR) using the Benjamini Hochenberg method (BHp-value) (**B**). BH p-values < 0.05 are considered significant. In addition, the overall mean signal intensity across all spots in the array, as well as the number of reactive HA strains, defined as those with median fluorescence >1000, are also determined (**C**). Inclusion of 3M-052 in either SE or Liposomal formulations resulted in an increased IgG2c binding levels, with significant increases in IgG2c median fluorescence observed in 3M-052 liposomal formulations. In addition, inclusion of 3M-052 resulted in a significant increase in overall mean signal intensity, as well as an increase in the number of positive spots and HA strains.

**Figure 4:**
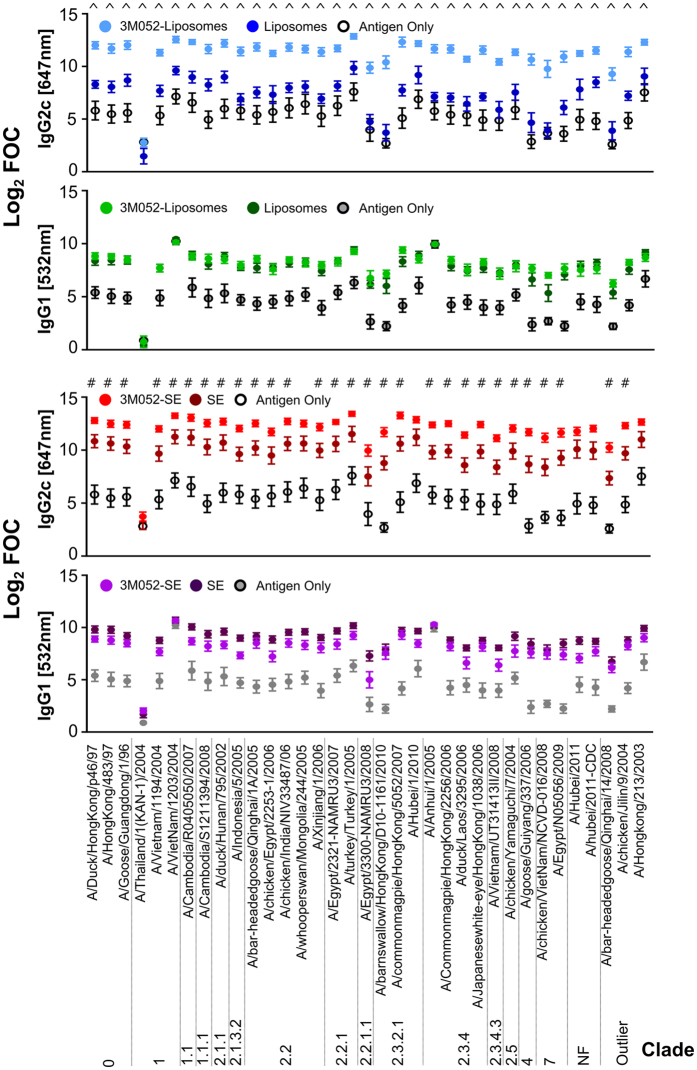
H5N1 Cross-Clade Antibody Binding Analysis of Sera From Mice Immunized With 3M-052 Adjuvants. Mice were immunized twice with split H5N1 vaccine (A/VN/1203/04, Sanofi Pasteur) 28 days apart (D0, D28) in combination with adjuvants as indicated. Adjuvant dose was 1 or 2 μg 3M-052 in SE or liposomes, respectively. Sera was collected from mice at D63 (n = 7/group), and analyzed using a high density HA array. Specific binding of both IgG1 and IgG2c antibodies to H5N1 proteins from different clades is shown. Specific binding levels are determined by normalization of array data to determine the fold over control (FOC) binding for each H5N1 protein. Log2 transformed FOC data for both SE and liposomal formulations with or without 3M-052 are presented for both IgG1 and IgG2c antibody subtypes with error bars representing standard error of them mean (SEM). Significant increases in specific binding resulting from the inclusion of 3M-052 are determined by calculation of Benjamini-Hochenberg FDR corrected P-values (Q = 5%), with BH p-vales < 0.05 considered significant. Significance was determined for 3M-052-Liposomes compared to liposomes (^), and 3M-052-SE compared to SE (#). Adjuvant formulations containing 3M-052 showed significantly higher specific binding of IgG2c antibodies across H5N1 clades, while significant increases in IgG1 binding were not detected.

Previous studies have investigated the ability of TLR agonist adjuvants to increase the breadth of antibody responses induced following immunization of mice. Results have shown that induction of a Th1-biased CD4+ T cell response results in increased in class switching of antibodies and subsequently increases in breadth and protective capacity^53^. Consistent with previous research, we observe an induction of Th1 CD4 T cells (IFNγ^+^TNFa^+^IL-2^+^) following immunization with formulated 3M-052 adjuvants (Supplementary Figure S3). In order to address the ability of 3M-052 adjuvant formulations to broaden the immune response to a pre-pandemic antigen, we have investigated the binding profile of 3M-052 to induced antibodies using a second generation high density HA protein array. The HA array used in this study contains 278 individual HA proteins, including at least one representative from HA subtypes 1–18. We have examined the ability of both IgG1 and IgG2c antibodies to bind to the array (Fig. 3, Supplementary Figure S4). Global profiling of antibody responses showed increased median fluorescence values for IgG2c antibodies in adjuvant formulations containing 3M-052 (Fig. 3B). These increases were significant (FDR adjusted p-value < 0.05) for IgG2c antibodies in the case of 3M-052/liposomes for H5N1 proteins as well as proteins from other HA subtypes. For 3M-052/SE, increases in IgG2c fluorescence were observed for many strains relative to SE based formulations, but these increases in median fluorescence were not significant, likely due to higher antibody binding levels observed in SE immunized animals compared with liposome formulations. As an additional assessment of the ability of adjuvant formulations to broaden antibody responses, we have determined the breadth, measured by the global signal intensity on the array across all spots, as well as the number of reactive strains, define as those with a signal intensity >1000, in the presence of adjuvant formulations (Fig. 3C). Addition of 3M-052 to SE or liposomal formulations resulted in a significantly increased global signal intensity (FDR adjusted p-value < 0.05), and an increase in the number of positive HA strains. In addition, through normalization of array data, we have examined the specific binding of antibodies induced by difference adjuvant formulations to H5N1 strains from diverse virus clades. Both 3M-052/liposome and 3M-052/SE immunized animals showed increased levels of IgG2c antibody that bound to proteins from multiple H5N1 virus clades (Fig. 4) compared with the same formulations without 3M-052. In contrast IgG1 binding levels were not enhanced by 3M-052 (Fig. 4). In order to verify the findings from the HA arrays, we determined antibody endpoint titer in post-immunization mouse serum by ELISA using VN1203 HA as a coating antigen (Supplementary Figure S5). The results observed by serum ELISA mirrored those observed on the HA array, consistent with previous data verifying protein structure on the array^54^.

### Formulated 3M-052 Adjuvants Enhance Protection Against H5N1 Challenge in Ferrets After a Single Immunization

**Figure 5:**
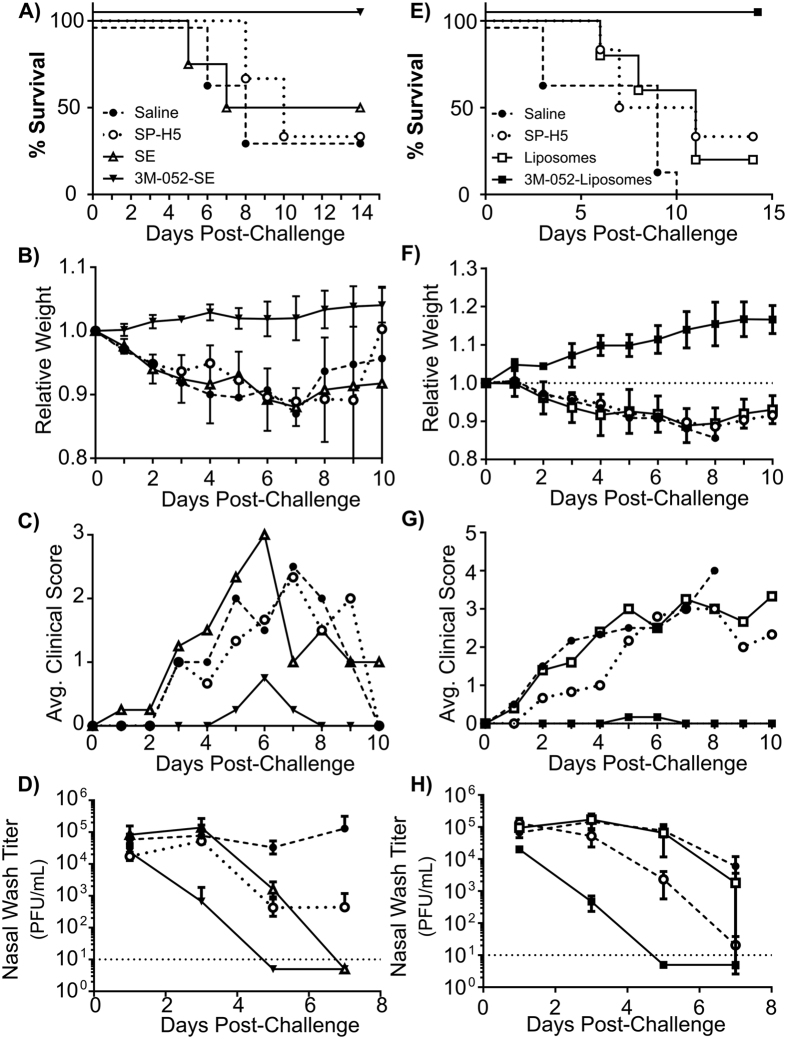
Protection of Ferrets From Homologous H5N1 Challenge Following A Single Immunization. Male Fitch ferrets (n = 3–4/group) were immunized once with a split H5N1 vaccine (H5N1, Sanofi Pasteur) in combination with formulated 3M-052 adjuvants (adjuvant dose was 1 or 2 μg 3M-052 in SE or liposomes, respectively), and challenged 21 days post immunization with 10^6^ PFU of A/VN/1203. Animals were monitored for up to 14 days for survival (**A**,**E**) weight loss (**B**,**F**) and clinical scores (**C**,**G**). In addition, nasal washes were collected to assess virus titer (**D**,**H**). For both weight loss and virus titer, points represent the arithmetic mean within the group, with error bars representing standard deviation of the mean. Both 3M-052/SE and 3M-052-Liposomal adjuvant formulations show potent single shot protection in this model, characterized by more rapid viral clearance from nasal washes, reduced weight loss and clinical scores, and 100% survival.

Given promising results in mice, we investigated the ability of formulated 3M-052 based adjuvants to protect ferrets from H5N1 challenge. Prior to initiation of these studies, we verified that the imidazoquinoline portion of 3M-052 could stimulate ferret TLR receptors. Using a whole blood stimulation assay, we verified that incubation with imiquimod, or CL057, which has a structure similar to 3M-052, could stimulate production of mRNA for cytokines and for receptors (Supplementary Figure S6). We then examined the ability of 3M-052 containing adjuvants to protect ferrets form lethal H5N1 challenge following a single immunization. For these studies, neutral liposome and SE adjuvants with and without 3M-052 were combined with an H5N1 split virus vaccine (Sanofi) derived from VN1203 (SP-H5). This antigen was chosen because it is currently included in the national pandemic stockpile for the United States, and has been tested clinically in previous studies. Briefly, following a dose ranging study with H5N1 antigen alone (Data not shown), groups of 4–6 ferrets were immunized via the intramuscular route with 0.5 μg of SP-H5 admixed with adjuvants as indicated (Fig. 5). Twenty-one days post-immunization, ferrets were challenged intranasally with 10^6^ PFU of A/VN/1203/04, and followed for 14 days for weight loss, clinical scores, and survival. In addition, nasal washes were collected on alternate days beginning one day post-challenge. In one study, 3M-052/SE completely protected animals from challenge, compared with animals immunized with SP-H5 + SE (Fig. 5A). 3M-052/SE also reduced virus-induced morbidity compared to SE alone; animals immunized with SP-H5 + 3M-052/SE lost no weight following challenge (Fig. 5B, Supplementary Figure S7), and had only minimal clinical scores throughout the infection (Fig. 5C). Similar results were observed in a second experiment following immunization of animals with liposomal adjuvant formulations. 3M-052/liposomes protected 100% of animals from challenge (Fig. 5E). Similar to SE based formulations, animals immunized with SP-H5 + 3M-052/liposomes lost no weight (Fig. 5F, Supplementary Figure S7), and had negligible clinical scores (Fig. 5G). Perhaps most importantly, examination of lung viral titers shows that in both SE and liposomal formulations, 3M-052 is capable of reducing virus shedding in nasal washes; animals immunized with 3M-052/SE (Fig. 5D) or 3M-052/liposomes (Fig. 5H), showed significant reductions as soon as 3 days post-challenge compared to other adjuvants or control animals. In both cases, virus was cleared to undetectable levels by day 5 in all animals receiving 3M-052 adjuvants.

### 3M-052 Adjuvant formulations induce a cross-clade neutralizing antibody response in ferrets

**Figure 6:**
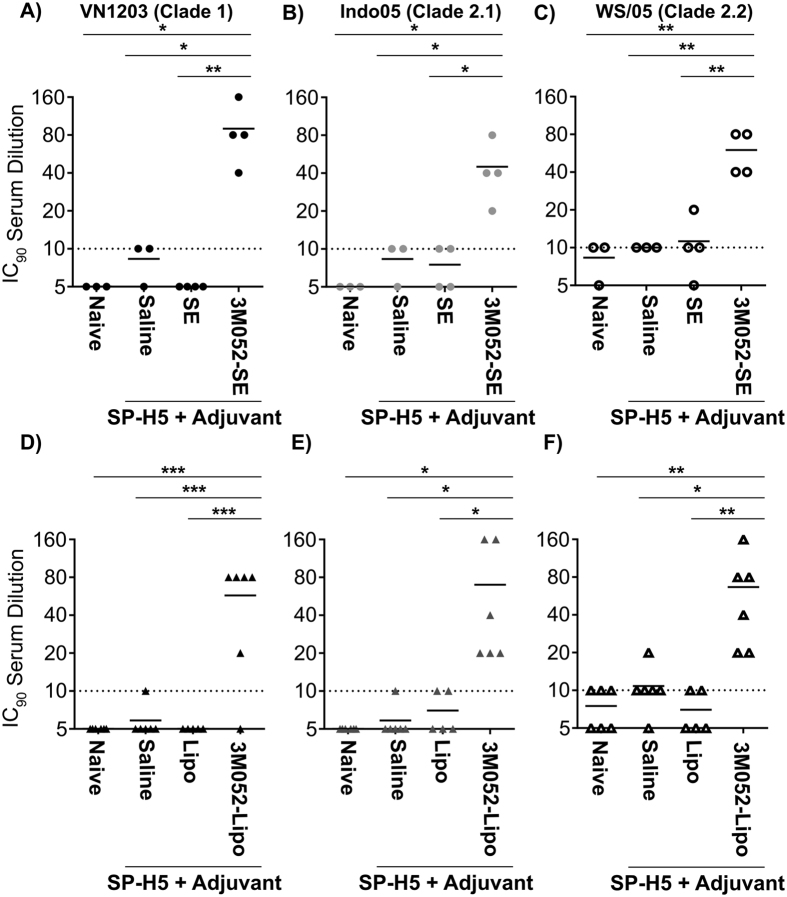
Induction of Virus Neutralizing Titer By Formulated 3M-052 Adjuvants. Male Fitch ferrets (n = 3–4/group) were immunized once with a split H5N1 vaccine (SP-H5, Sanofi Pasteur) in combination with formulated 3M-052 adjuvants. Adjuvant dose was 1 or 2 μg 3M-052 in SE or liposomes, respectively. Twenty one days post-immunization, blood was collected from all animals, and assayed for virus neutralizing antibodies using a retrovirus pseudotype neutralization assay. Inclusion of 3M-052 in adjuvant formulations resulted in significant (one-way ANOVA) increases in neutralizing titer against both homologous clade 1 virus (**A**,**D**) as well as a clade 2 virus strain (**B**,**E**) and a Clade 2.2 strain (**C**,**F**).

Based on the broad HA-specific antibody response that we observe in mice and the robust protection we observed against homologous virus in ferrets, we directly examined the neutralizing antibody titers induced following immunization with different adjuvant formulations using a H5N1 HA pseudotyped lentivirus vector packaging a Luciferase transgene. Briefly, serially diluted serum samples were incubated with lentiviral particles containing H5N1 HA and NA proteins on their surface. Serum-virus mixtures were incubated on confluent monolayers of MDCK cells. Following incubation to allow expression of the luciferase transgene packaged into the virus, neutralization potential of serum samples was determined by quantification of luciferase gene expression. Consistent with increased survival observed in these studies, 3M-052/SE and 3M-052/liposomes adjuvants increased IC_90_ neutralization titers in serum 21 days following a single injection. (Figure 6A,D). In addition, both formulations significantly increased neutralization titers to Clade 2 viruses; increased IC_90_ titers to A/Indo/5/05 [Clade 2.1] and A/Whooper swan/Mongolia/244/05 [Clade 2.2] were observed (Fig. 6B–F). This finding is consistent with the ability of formulated 3M-052 to induce a broadened functional neutralizing antibody titer to drifted influenza strains.

### 3M-052/SE Induces a Cross-clade Protective Response

**Figure 7:**
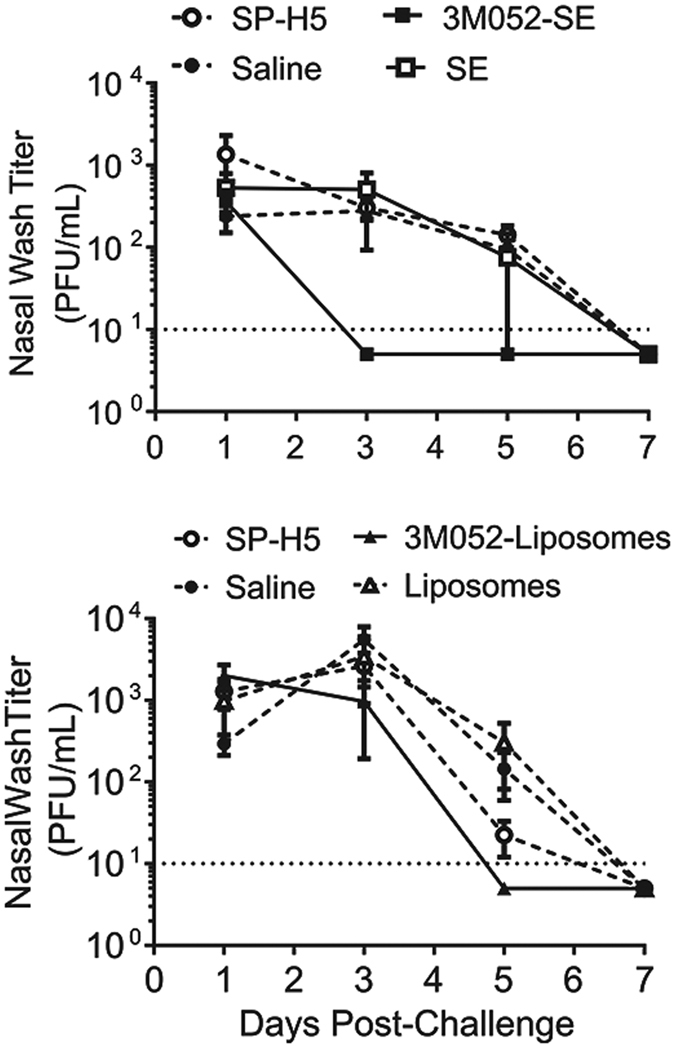
Protection of Ferrets From Heterologous H5N1 Challenge Following A Single Immunization. Male Fitch ferrets (n = 6/group) were immunized once with a split H5N1 vaccine (H5N1, Sanofi Pasteur) in combination with formulated 3M-052 adjuvants (adjuvant dose was 1 or 2 μg 3M-052 in SE or liposomes, respectively), and challenged 21 days post immunization with 10^6^ PFU of A/Whooper Swan/Mongolia/244/05. Nasal washes were collected to assess virus titer. 3M-052/SE adjuvants induced a more rapid clearance of virus, with undetectable titers at day 5. 3M-052-Liposomal adjuvant formulations show titer through day 3, with clearance by day 5.

Given the neutralizing titers that we observe to A/Whooper swan/Mongolia/244/05 following immunization with VN1203 antigens, we investigated the ability of formulated 3M-052 to reduce virus replication in the lung following vaccination with VN1203 antigen. As in the previous studies, ferrets were immunized once with SP-H5 combined with formulated 3M-052, and challenged 21 days later with 5 × 10^5^ PFU of virus. Animals receiving 3M-052/liposomes showed a modest decrease in virus shedding over time; animals receiving 3M-052/liposomes had no detectable virus at 5 days post-challenge, while other animals receiving liposomes or antigen alone had detectable virus at this time (Fig. 7B). In contrast, animals receiving 3M-052/SE rapidly cleared the virus, with no detectable plaques from 3 days post challenge, while those receiving SP-H5 + SE showed detectable virus titers through day 5 (Fig. 7A).

## Discussion

The continued circulation of H5N1 HPAI viruses in wild-birds and domestic poultry and their ability to transmit sporadically into the human population indicates that viruses of this subtype continue to pose a pandemic threat. Given the sporadic emergence of the virus into humans from multiple avian reservoirs, the generation of a strain-matched vaccine during the early stages of an emerging pandemic is unlikely. The majority of pandemic preparedness plans call for stockpiling of pre-pandemic H5N1 vaccines in the hope that these antigens will provide sufficient protection to limit mortality in the event of a pandemic^11^. As the diversity of circulating H5N1 clades has risen, there is increasing demand for adjuvants which are compatible with stockpiled H5N1 vaccines, and which may broaden the immune response elicited by these vaccines in order that they will generate protective responses to heterologous strains. The number of adjuvants licensed for use in humans is relatively small. Alum based adjuvants, which are historically widely used, are ineffective when combined with H5N1 influenza vaccines^55^. More recently, squalene oil-in-water adjuvants have been tested extensively with H5N1 vaccines, and have demonstrated critical dose sparing as well as broadening functions^33^,^34^,^56^.

The results presented here conclusively demonstrate protection in mice and ferrets following high dose (1000 LD_50_) challenge with H5N1 viruses when animals are immunized once with the formulated TLR7/8 agonist 3M-052. 3M-052 is incorporated into either liposomal or emulsion based formulations, and these formulations are stable for extended periods. A novel PEGylated liposome formulation of 3M-052 was observed to increase survival and decrease lung virus titer when combined with a low dose (100 ng) of a recombinant HA based antigen. In addition, we have tested a novel emulsion based formulation which incorporates 3M-052 into a squalene oil-in-water emulsion. Our findings, which are consistent with previous published work, show that SE based adjuvant formulations enhance protection to homologus antigen in mice; we observed protection of 100% of animals from virus induced mortality. However, examination of lung titers, as well as D7 lung weights and pathology scores in these animals show that addition of 3M-052 to SE based formulations significantly reduces lung virus titers, which indicates an increased capacity of 3M-052 induced antibodies to neutralize virus and inhibit spread. Furthermore, our studies suggest that co-localization of the TLR agonist with the emulsion based formulation post-injection is critical for the induction of antibodies which reduce viral replication in the lung. Consistent with previous studies, which show that small molecule agonists such as R848 diffuse away from injection sites, R848 combined with emulsion based adjuvants did not reduce lung viral titer post-immunization, and mean titers were similar to those observed with SE alone (Fig. 2). In contrast, animals immunized with 3M-052/SE show significant reductions in virus titer relative to animals immunized with SE. and have shown increased survival and reduced lung titer with this formulation in combination with rHA.

The ability of adjuvants to stimulate cross-clade protection against diverse H5N1 strains is of critical importance in the development of effective adjuvants for pandemic vaccines. In order to assess the potential of 3M-052 to broaden the antibody responses, we examined the binding profile of post-immunization serum using a high-density HA protein array. In this assay, we define broadening as an increase in the number of HA strains which are recognized by antibodies induced by different adjuvant formulations. We find that 3M-052, formulated in either SE or liposomal formulation, results in a statistically significant increase in the mean signal intensity across all spots on the array by IgG2c antibodies (Fig. 3C). Similar increases in signal intensity were not observed with IgG1 antibodies. An examination of the number of strain on the array which are positive in difference adjuvant groups is consistent with a broader immune response, as opposed to high level binding to homologous antigen. Detailed examination of the H5N1 strains on the array shows that the specific binding of IgG2c antibodies to a variety of H5N1 strains from different virus clades is significantly enhanced by 3M-052 in both SE and liposomal formulations (Fig. 4).

The appearance of a broadly cross-reactive antibody response in mice prompted us to expand our studies to the ferret challenge model. We have confirmed that imidazoquinolines can stimulate ferret TLR receptors, and observe significant increases in both cytokine and TLR7 and TLR8 mRNA following incubation with imidazoquinolines. As in mice, immunization of ferrets with a single dose of H5N1 antigen induced functional protection against both homologous (Fig. 5) and heterologous virus strains (Fig. 7). The protection against heterologous strains is consistent with the induction of cross reactive neutralizing antibody responses in ferrets by formulated 3M-052 adjuvants.

The ability of 3M-052 to broaden antibody responses, ultimately allowing protection from heterologous virus challenge, could be attributed to a global increase in antibody levels stimulated by the adjuvant, or to changes in the antibody repertoire induced in the presence of the TLR agonist, such that antibodies with novel variable regions with diverse binding specificities are induced. While our studies conclusively demonstrate an enhanced protective capacity of antibodies induced by 3M-052/SE and 3M-052/Liposomes, it is unclear whether this is due to induction of unique antibody sequences. Our previous work has demonstrated the ability of 3M-052 to increase IgG V-gene diversity following delivery with a malarial antigen. In this case, an increase in the number of IgG variable region sequences was observed following immunization of mice^53^. Future studies to specifically examine the ability of these adjuvants to increase antibody sequence diversity in the context of H5N1 influenza antigens may provide a potential mechanism for the increased protective capacity observed in our studies, and would augment the binding analysis conducted here. This assessment may be particularly important in addressing the suitability of 3M-052-SE or 3M-052-Liposomes for use in broadly protective “universal” influenza vaccine approaches. We have conducted preliminary investigation into the ability of the serum generated in these studies to neutralize HA proteins from other subtypes, most notably A/California/4/2009 (H1N1), and have not observed neutralization *in vitro*. However, our array data may suggest additional strains which may be selected to further examine the functional breadth of responses induced by 3M-052.

Adjuvant formulations containing innate immune stimulants including TLR agonists and RIG-I activating compounds have previously been shown to broaden antibody responses and enhance protection to heterologous virus strains^57^,^58^,^59^. Here, we report the development of additional formulations which are capable of inducing broadly protective and functional antibody responses. In addition, we have previously shown that the TLR4 agonist GLA, formulated either in a stable emulsion^46^ or as an aqueous formulation^45^, was shown to protect both mice and ferrets against both homologous and heterologous H5N1 challenge. These formulations have also been tested clinically via both intramuscular and intradermal routes (NTC01397604, NCT02465216, NCT01991561, NCT01147068). The safety profile of formulated TLR4 agonists is promising, and this opens up the possibility of synergistic agonist formulations containing both agonist 3M-052 and GLA molecules. TLR combination adjuvants have been tested in vaccines for a number of disease indications including influenza^57^ and have shown promise. However, many of these pre-clinical studies use TLR-agonist formulations which are impractical for advancement into the clinic due to lack of a clinically viable formulation^60^.

In conclusion, the data presented here demonstrate the utility of 3M-052 as an adjuvant for pandemic influenza vaccines in multiple pre-clinical models. The ability of 3M-052 containing adjuvant formulations to induce protection against both homologous and heterologous virus strains, as well as the extended stability of formulated 3M-052 adjuvants suggests that these formulations are suitable for stockpiling. Furthermore, we have demonstrated compatibility of these adjuvants with the pre-pandemic vaccine antigens currently included in the national stockpile, and show that a combination vaccine is capable of protecting ferrets against both strain-matched and drifted isolates. Future studies will be directed at cGMP manufacturing of 3M-052 adjuvant formulations, and advancement into toxicology and Phase I clinical testing.

## Materials and Methods

### Formulation of materials and manufacture

3 M-052 was synthesized in-house by the 3 M Company and its structure has been previously published^50^. R848 was supplied by 3 M or purchased from Axxora Life Sciences Inc. (San Diego, CA). Synthetic 1,2-dipalmitoyl-sn-glycero-3-phosphocholine (DPPC), 1,2-dimyristoyl-sn-glycero-3-phosphocholine (DMPC), 1,2-dioleoyl-sn-glycero-3-phosphocholine (DOPC), 1,2-dihexadecanoyl-sn-glycero-3-phospho-(1′-rac-glycerol) (DPPG), 1,2-dipalmitoyl-3-trimethylammonium-propane (DPTAP), 1,2-dipalmitoyl-sn-glycero-3-phosphoethanolamine-N-[methoxy(polyethylene glycol)-750] (DPPE-PEG750), and egg phosphatidylcholine (PC) were purchased from Avanti Polar Lipids Inc. (Alabaster, AL) or Lipoid LLC (Newark, NJ). Squalene was obtained from Sigma (St. Louis, MO). Cholesterol, ammonium phosphate monobasic, and ammonium phosphate dibasic were purchased from J.T. Baker (San Francisco, CA). Poloxamer 188 and glycerol were purchased from Spectrum Chemical (Gardena, CA). Phosphate buffered saline 1 × (PBS) at pH 7.2 was purchased from Invitrogen (Grand Island, NY).

Small batches (≤20 mL) of PEGylated liposome formulations were manufactured by combining DPPC, cholesterol, and DPPE-PEG750 with various amounts of 3M-052 in organic solvent (chloroform or chloroform/methanol/water mixture). The organic solvent was then evaporated using a Genevac EZ-2. The lipid film was rehydrated in PBS (pH 7.2) or 25 mM ammonium phosphate buffer (pH 5.7) and sonicated in a Crest powersonic CP230D (Trenton, NJ) sonicating water bath at ~60 °C for ~2–3 hrs or until the formulation was translucent with no large visible particles. The same procedure was followed for neutral liposomes (DOPC, cholesterol), anionic liposomes (DPPC, cholesterol, DPPG), and cationic liposomes (DPPC, cholesterol, DPTAP). The liposome component weight ratios were as follows: PEGylated (18:5.5:3, DPPC:chol:DPPE-PEG750), neutral (20:5, DOPC:chol), anionic (18:5:2, DPPC:chol:DPPG), cationic (18:5:2, DPPC:chol:DPTAP). Larger batches (100 mL) of PEGylated liposomes were manufactured as above but with shorter sonication time followed by high shear homogenization (Microfluidizer M110P) for 12 continuous passes at 30,000 psi.

Liposomes containing R848 were prepared by manufacturing a concentrated PEGylated liposome composition as described above except that the liposomes were initially hydrated with 75 mM ammonium sulfate solution. Following sonication at 60 °C ~1 h, a PD-10 column (GE Healthcare) was employed to exchange the external buffer of the liposomes to 0.9% saline. The liposomes, now containing saline in their external buffer and ammonium sulfate in their interior, were mixed with a saline solution containing R848 and incubated for 1 h at 60 °C. Finally, the liposomes were passed through another PD-10 column to remove unencapsulated R848.

Oil-in-water stable emulsions (SE) were manufactured by dissolving 3M-052 into chloroform with egg PC. The chloroform was then removed using a rotary evaporator. Squalene was then added to the dried lipid film and the glass container was placed in a 60 °C sonicating water bath for ~1 h. This mixture is referred to as the oil phase. Alternatively, the oil phase was prepared by dispersing 3M-052 directly into squalene and DMPC (no egg PC or chloroform). An aqueous phase was then added to the oil phase to obtain final concentrations of 25 mM ammonium phosphate buffer, 0.037% (w/w) poloxamer 188, and 1.8% (v/v) glycerol (isotonic agent), 4% v/v squalene, 7.6 mg/ml egg PC or DMPC, and various amounts of 3M-052. Some emulsions also contained 0.02% v/v α-tocopherol. The crude emulsion was created by sonicating the mixture in the 60 °C water bath for another 10–15 minutes. The final emulsion was prepared by processing the crude emulsion through a high shear homogenizer (Microfluidizer M110P) for ~12 continuous passes at 30,000 psi.

Emulsions were prepared with R848 by manufacturing a concentrated emulsion as described above and mixing with dry powder R848 or with a solution of R848 in ammonium phosphate buffer. Emulsions and liposomes were filtered through a 0.2 μm membrane prior to the *in vivo* experiments described below.

### Formulation characterization

Concentrations of 3M-052 and R848 were estimated by first diluting each formulation 20-fold into a 98% ethanol/2% HCl solution in a cuvette. The samples were analyzed on a Hitachi U-3900H spectrophotometer (Tokyo, Japan) for absorbance at ~322 nm. Particle size was evaluated using a Malvern Instruments (Worcestershire, UK) Zetasizer Nano-S, -ZS, or -APS. Formulations were diluted 100-fold into ultrapure water in a 1.5 ml polystyrene disposable cuvette. For each formulation, three separate cuvettes were prepared. All size measurements were then made three times for each cuvette. Zeta potentials were measured using the Malvern Zetasizer Nano-ZS. For each formulation, 50 μl were combined with 950 μl ultrapure water in a disposable capillary cell (Malvern Instruments, DTS1070). Nine measurements were collected from each prepared sample (one sample per formulation).

### Virus Stocks and Vaccines

Vaccine antigens used in this study were all derived from the A/Vietnam/1203/04 (VN1203) influenza virus. Recombinant HA protein (rHA) used in murine immunogenicity and challenge studies was obtained from Protein Sciences Corp. For ferret immunization and challenge studies, a clinical grade split VN1203 vaccine was obtained from Sanofi Pasteur.

Virus stocks for murine and ferret challenge studies were generated by inoculation of 10-day old embryonated chicken eggs with H5N1 stocks as indicated. Clarified allantoic fluid was filtered, and stored at −80 °C until use. Viral titers were determined by plaque assay on MDCK cells (ATCC #CCL-34) using standard assays and as described.

### Animal Studies

All murine immunogenicity studies were carried out in the IDRI animal care facility (Seattle, WA) under specific pathogen-free conditions, and in accordance with previously approved techniques. All animal experiments and protocols used in this study were specifically approved by IDRI’s Institutional Animal Care and Use Committee (IACUC). Murine challenge studies were carried out by contract with the National Institutes of Health at the University of Utah. Experimental protocols for this work were specifically approved by the IACUC. Ferret challenge studies were carried out in the ABSL3 facility at Colorado State University (CSU) in accordance with established experimental procedures, and were specifically approved prior to initiation by the CSU IACUC.

### Murine Challenge Studies

For murine challenge studies groups of 6–8 week old female C57Bl/6 mice (n = 10/group) were immunized with recombinant H5N1 HA protein (VN1203 strain, Protein Sciences Corp.) combined with adjuvant as indicated. All immunizations were via the intra-muscular route in a total volume of 100 μL split evenly between both legs. Twenty one days following immunization, mice were challenged with 1 × 10^6^ PFU of A/VN/1203/05 (H5N1 Clade 1). Beginning one day post-challenge, all animals were monitored for weight loss and observed for signs of severe infection. Animals found to have lost >25% of day 0 weight were euthanized.

### Ferret Challenge Studies

Male Fitch ferrets (*Mustela putoris fero*, Triple F Farms, Sayre PA) were used for all immunization and challenge studies. Immunization of ferrets was carried out by injection of 250 μL into the quadriceps muscle. All animals received 0.5 μg of a split A/Vietnam/1203/04 H5N1 vaccine (Sanofi Pasteur) sourced from the national pandemic stockpile. Serum samples were collected 21 days post-immunization to determine antibody titers.

Challenge was initiated by instillation of 1 × 10^5^–5 × 10^5^ PFU of virus into the lung in a volume of 500 μL (250 μL/nare). Following infection, animals were observed and weighed daily to monitor virus induced morbidity, and nasal washes were collected under ketamine-xylazine sedation on days 1, 3, 5, 7, and 9 post-challenge. Any animal displaying severe clinical signs or losing >25% of pre-challenge weight was euthanized.

### Plaque Assay

Virus titers were obtained by plaque assay in Madin-Darby canine kidney (MDCK) cells. Briefly, cells were seeded 24 hours prior to assay initiation into 6-well dishes at 1 × 10^6^ cells/well in Dulbecco’s modified Eagle’s medium (DMEM) supplemented with 10% fetal bovine serum (FBS) and penicillin/streptomycin. At assay initiation, confluent MDCK monolayers were washed three times with PBS to remove FBS. Serially diluted nasal wash samples were added to monolayers in a 100 μL volume and incubated at 37 °C for 60 minutes. Following incubation, monolayers were overlaid with 2 mL of 0.8% Agarose (SeaKem) in MEM(Lonza). Following 48–72 hours of incubation, plaques were quantified following crystal violet (BD) staining.

### Microneutralization Assay

Neutralizing antibody titers were determined using pseudotyped lentivirus particles similar to those previously described^61^,^62^. Briefly, pseudotype particles are generated by co-transfection of 293 T cells with plasmids expressing H5N1 and HA, and 3 lentivirus packaging plasmids; pDR8.74^63^, pRSV-Rev, and HR’-CMV-Luc, which is packaged into the viral particles and encodes a luciferase transgene under the control of the CMV immediately early promoter. Virus stocks were titred in black flat-bottom 96 well plates (Corning) seeded 24 hour prior with 5 × 10^3^ MDCK cells/well in Dulbecco’s modified Eagle’s medium (DMEM) + 10% fetal bovine serum (FBS). Prior to virus titration, cells were washed 3 times with 200 μL DMEM to remove DMEM, and overlaid with serially diluted virus stocks. Following 72 hour incubation to allow transgene expression, levels of luciferase in transduced cells were determined using BrightGlo luciferase (Promega), according to manufacturer’s instructions.

For neutralization analysis, serially diluted post-immunization serum was mixed at a 1:1 ratio with diluted virus stocks containing 1 × 10^4^ relative luciferase units (RLU). Virus serum mixtures were incubated at 37 °C for 1 hour, and added to plates containing washed MDCK cells as above. Neutralization titers were determined following assessment of luciferase expression level in all transduced cells. IC_90_ antibody titer is defined as the highest dilution of serum observed to reduce luciferase levels by 10-fold relative to vector transduced control cells.

### HA Arrays

HA Arrays containing influenza HA proteins have been previously described^54^. The arrays used in this study are second generation, and contain 278 recombinant HA proteins from a variety of influenza strains (Sinobiological). HA proteins from each strain are spotted in the array at 3 different concentrations (0.1, 0.03, 0.01 pg/spot) to allow dose response analysis. For post-vaccination immune analysis, arrays were initially blocked with PBS + 1% Fetal Bovine Serum + 0.1% Tween-20, washed three times with Protein Array Wash Buffer (ArrayIt), and incubated with 300 μL of a 1:100 dilution of post-immunization mouse serum for 1 hour with shaking. Following primary incubation, arrays were washed 5 times with wash buffer, and incubated with fluorophore conjugated secondary antibodies for IgG2c (Jackson Immunoresearch, Part #: 115-495-208) and IgG1 (Life Technologies, Part#: A21123) at a 1:2000 dilution. Following a 30 minute incubation at room temperature, arrays were rinsed with Rinse Buffer (Arrayit), and analyzed using a Molecular Dynamics 400B array scanner.

### Statistical Analysis of Array Data

Background signals from sample specific median for negative control spots (PBS buffer) were subtracted from purified protein spot signals. The data were then normalized by dividing the background removed purified protein spot intensity by the global median for the negative control spots throughout the chip (fold over control [FOC]) and then taking the base 2 logarithm of the ratio (log_2_ FOC). The normalized data provide a relative measure of specific antibody binding to nonspecific antibody binding to the purified protein controls (i.e., the background). The base stats package in R was used to calculate 2-sided *t* test *P* values on log2 transformed data and to correct *P* values for false discovery using the Benjamini-Hochberg method^64^. Differences were considered significant with Benjamini Hochberg corrected *p*-values < 0.05. Antigens with average signals above 1000 within each group were considered to be reactive antigens due to low background signals from PBS buffer. To compare multiple groups, Kruskal–Wallis/Dunn multiple-comparison tests with corrected *P* values were calculated in R.

### ELISA

HA-specific endpoint titers for IgG, IgG1 and IgG2c were determined seven days and twenty-one days post immunization. High binding polystyrene 384 well plates were coated with recombinant VN1203 HA (Protein Sciences Corp.) (2 μg/ml) in 0.1 M bicarbonate coating buffer for 2.5 hours at room temperature. Plates were washed three times with 0.1% PBS–Tween 20 pre and post a two hour blocking incubation with 0.05% PBS–Tween 20 + 1% BSA at room temperature. Mouse sera was serially diluted in 0.05% PBS–Tween 20 + 0.1% BSA using the Nanonscreen NSX-1536 and incubated overnight at 4 °C and washed five times. Plates were incubated for 1 hour on the shaker with anti-mouse IgGT, IgG1 or IgG2c-HRP (Southern Biotechnologies). Following five washes, plates were developed on the Nanoscreen robot using SureBlue tetramethylbenzidine substrate (Kirkegaard & Perry Laboratories). The enzymatic reaction was stopped with 1 N H_2_SO4 using the Multipette Sagian robot. Plates were read at 450–570 nm using the Synergy ELISA plate reader (Biotek) and Gen 5 software.

## Additional Information

**How to cite this article:** Van Hoeven, N. *et al*. A Formulated TLR7/8 Agonist is a Flexible, Highly Potent and Effective Adjuvant for Pandemic Influenza Vaccines. *Sci. Rep.* **7**, 46426; doi: 10.1038/srep46426 (2017).

**Publisher's note:** Springer Nature remains neutral with regard to jurisdictional claims in published maps and institutional affiliations.

## Supplementary Material

Supplementary Data

## Figures and Tables

**Figure 1:**
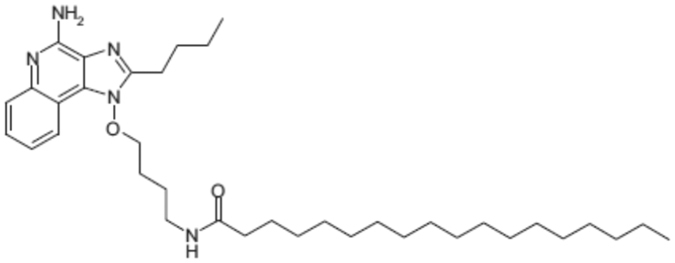
Chemical structure of 3M-052.

**Table 1:** Formulation properties of liposome and emulsion formulations of 3M-052.

Formulation Type*	Composition	Batch #1	Batch #2
		Size (Z-Ave, nm), PdI, and Appearance	Size (Z-ave, nm), PdI, and Appearance
Anionic Liposome	DPPC (18 mg/mL), DPPG (2 mg/mL), cholesterol (5 mg/mL)	155.8, 0.398, opaque/milky	155.9, 0.716, opaque/milky
Cationic Liposome	DPPC (18 mg/mL), DPTAP (2 mg/mL), cholesterol (5 mg/mL)	145.6, 0.539, translucent	210.7, 0.560, translucent
PEGylated Liposome	DPPC (18 mg/mL), DPPE-PEG750 (3 mg/mL), cholesterol (5.5 mg/mL)	143.1, 0.284, translucent	90.9, 0.185, translucent
Neutral Liposome	DOPC (20 mg/mL), cholesterol (5 mg/mL)	123.5, 0.354, translucent	44.0, 0.146, translucent
Oil-in-water emulsion (SE)	Squalene (34 mg/mL), egg PC or DMPC (7.6 mg/mL), poloxamer 188 (0.36 mg/mL), glycerol and ammonium phosphate buffer	91.2, 0.056, opaque/milky	83.9, 0.048, opaque/milky

^*^Liposomes and emulsions shown here were manufactured to contain 1 mg/ml 3M-052 or 0.04 mg/ml 3M-052, respectively, and the indicated excipient concentrations. Subsequent liposome compositions for stability and *in vivo* testing were manufactured to contain 0.04 mg/mL 3M-052, 7.2 mg/mL DPPC, 1.2 mg/mL DPPE-PEG750, and 2.2 mg/mL cholesterol.
