# Supplementary material for: A Formulated TLR7/8 Agonist is a Flexible, Highly Potent and Effective Adjuvant for Pandemic Influenza Vaccines
Source: Sci Rep. 2017 Apr 21;7:46426. doi: 10.1038/srep46426 (PMC5399443; doi:10.1038/srep46426)
Supplement: Supplementary Data [file srep46426-s1.pdf]

**Supplementary Material:**

# A Formulated TLR7/8 Agonist is a Flexible, Highly Potent and Effective Adjuvant for Pandemic Influenza Vaccines.

Authors: Neal Van Hoeven[1*], Christopher B. Fox[1], Brian Granger[1], Tara Evers[1], Sharvari W. Joshi[1], Ghislain I. Nana[1], Sarah C. Evans[1], Susan Lin[1], Hong Liang[1], Li Liang[4], Rie Nakajima[4], Phillip Felgner[4], Richard A. Bowen[2], Nicole Marlenee[2], Arin Hartwig[2], Susan L. Baldwin[1], Rhea N. Coler[1], Mark Tomai[3], James Elvecrog[3], Steven G. Reed[1], Darrick Carter[1].

[1]Infectious Disease Research Institute, 1616 Eastlake Ave E., Seattle WA 98103, USA,

[2]Colorado State University Department of Biomedical Sciences, Foothills Campus, Fort Collins, CO 80523, USA.

[3]3M, Inc., St. Paul, Minnesota 55121, USA.

[4]University of California Irvine, Department of Medicine, Irvine CA 92697, USA.

* Corresponding Author: neal.vanhoeven@idri.org

## *Supplementary Methods:*

### Ferret Whole Blood Stimulation Assay:

Whole blood collected from male Fitch ferrets was collected and incubated with TLR agonist compounds including Imiquimod (TLR7), the TLR7/8 agonist CL057 (Invivogen), and the synthetic TLR4 agonist GLA. Following incubation, RNA was collected from both stimulated and saline controls using an RNA whole blood extraction kit (Qiagen). Levels of RNA for TLR7, TLR8, IL-1β, and IL-8 were determined by real time PCR using established protocols [1]. Expression level changes were graphed as fold change in RNA levels relative to saline stimulated blood samples.

**Intracellular cytokine staining**

In order to quantify vaccine specific T cell responses, splenocytes were isolated from five mice per group following vaccination. Red blood cells were lysed using Red Blood Cell Lysis Buffer (eBioscience) and resuspended in cRPMI 1640(10% FBS, 1% Penicillin/Streptomycin; 0.1% 2-Mercaptoethanol). Cells were plated at $10^7$ cells/well in 96-well plates and were stimulated for 2 hours with media or rHA (10 µg/mL) at 37˚C. 1:50 GolgiPlug (BD Biosciences) was added and the cells were incubated for an additional 8 hours at 37˚C. Cells were washed and surface stained with fluorochrome labeled antibodies at 1:100 in 1%BSA-PBS to CD4 (clone RM4-5), CD8 (clone 53-6. 7), CD44 (clone IM7) and B220 (RA3-6B2) (BioLegend and eBioscience) in the presence of anti-CD16/32 (clone 93) for 15 minutes in the dark at room temperature. Cells were fixed and permeabilized with Cytofix/Cytoperm (BD Biosciences) for 30 minutes at room temperature in the dark. Cells were washed with Perm/Wash (BD Biosciences) and stained with fluorochrome labeled antibodies to detect intracellular cytokines as follows: IFN-γ (clone XMG-1.2), IL-2 (JES6-5H4), TNF (MP6-XT22), IL-5 (clone: TRFK5) and IL-10 (clone: JES5-16E3) (BioLegend and eBioscience) Staining was carried out for 15 minutes at room temperature in the dark. Cells were washed, resuspended in 1% BSA-PBS and filtered using a 30-40 µm PP/PE 96 filter plate (Pall Corp). Up to $10^6$ events were collected on a four laser LSR Fortessa flow cytometer (BD Biosciences). Data were analyzed with FlowJo (Treestar).

**References:**

1 Carolan, L. A. *et al.* TaqMan real time RT-PCR assays for detecting ferret innate and adaptive immune responses. *J Virol Methods* **205C**, 38-52, doi:10.1016/j.jviromet.2014.04.014 (2014).

***Supplementary Tables***

**Supplementary Table T1**. Zeta potentials of representative liposome and emulsion formulations containing 3M-052.

| **Formulation** | **Estimated 3M-052 Concentration (mg/ml)*** | **Zeta Potential (mV)** |
|---|---|---|
| PEGylated Liposome | - | -37.1 ± 4.8 |
| PEGylated Liposome | 0.04 | -27.1 ± 1.1 |
| Oil-in-water emulsion | - | -4.2 ± 0.6 |
| Oil-in-water emulsion | 0.04 | -8.6 ± 1.0 |

*3M-052 content was not measured for these batches but was estimated to be 0.04 mg/ml based on subsequent batches manufactured using the same process.

**Supplementary Table T2**. 3M-052 recovery following emulsion manufacture.

| Emulsifier | Premixed in $CHCl_3$ (Y/N) | Target 3M-052 Concentration (mg/ml) | Measured 3M-052 Concentration Pre-Filtration (mg/ml) | Measured 3M-052 Concentration Post-Filtration (mg/ml) | 3M-052 Recovery |
|---|---|---|---|---|---|
| DMPC | N | 0.12 | -- | 0.01 | 8% |
| DMPC | N | 0.12 | -- | <0.01 | <8% |
| DMPC | N | 0.04 | -- | 0.01 | 25% |
| DMPC | N | 0.04 | -- | <0.01 | <25% |
| DMPC | Y | 0.12 | 0.09 | 0.04 | 33% |
| Egg PC | N | 0.12 | 0.08 | 0.05 | 42% |
| Egg PC | Y | 0.12 | 0.12 | 0.12 | 100% |
| Egg PC | Y | 0.08 | 0.08 | 0.08 | 100% |
| Egg PC | Y | 0.8 | 0.76 | 0.32 | 40% |

***Supplementary Figures***

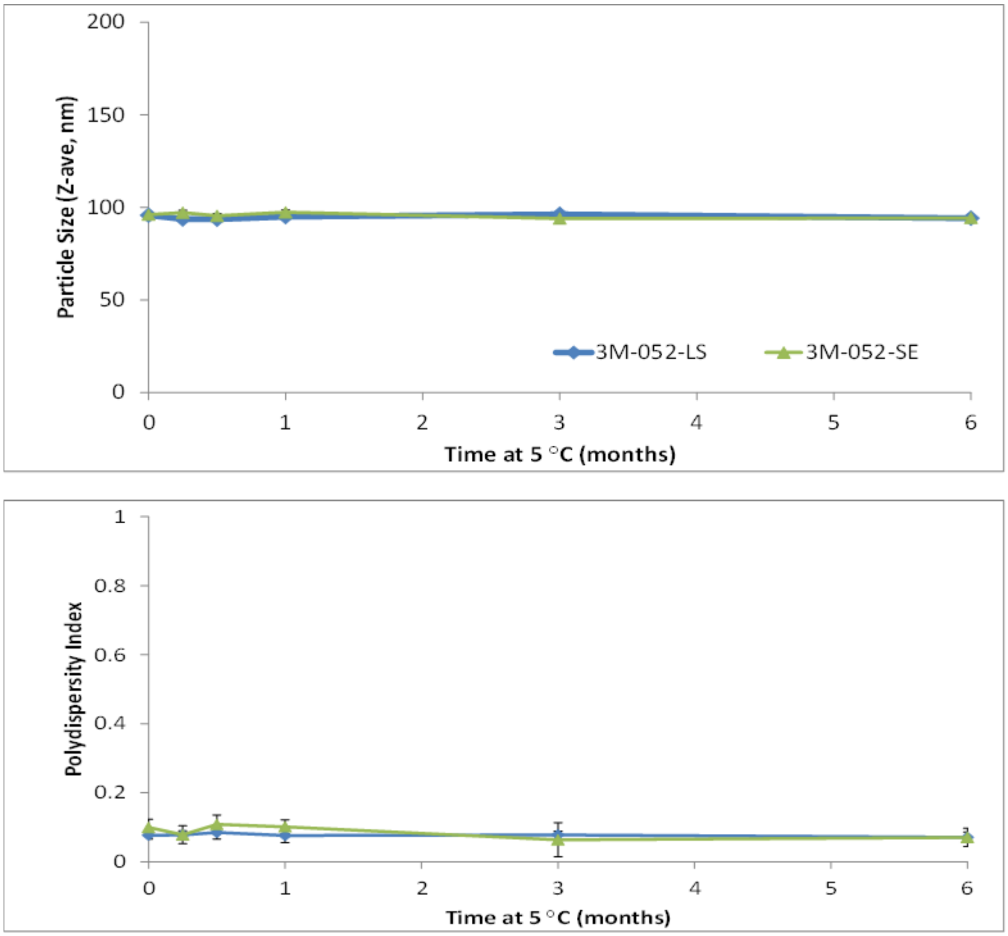


**Supplementary Figure S1. Liposomal and oil-in-water emulsion formulations of 3M-052 demonstrate long-term particle size stability.**

(a) Particle diameter and (b) size polydispersity index of representative lipid-based formulations of 3M-052 at 5°C for 6 months. Error bars represent standard deviation of three separate particle size assays from a single formulation batch at each timepoint. 3M-052 content was not measured for these batches but was estimated to be 0.04 mg/ml based on subsequent batches manufactured using the same process.

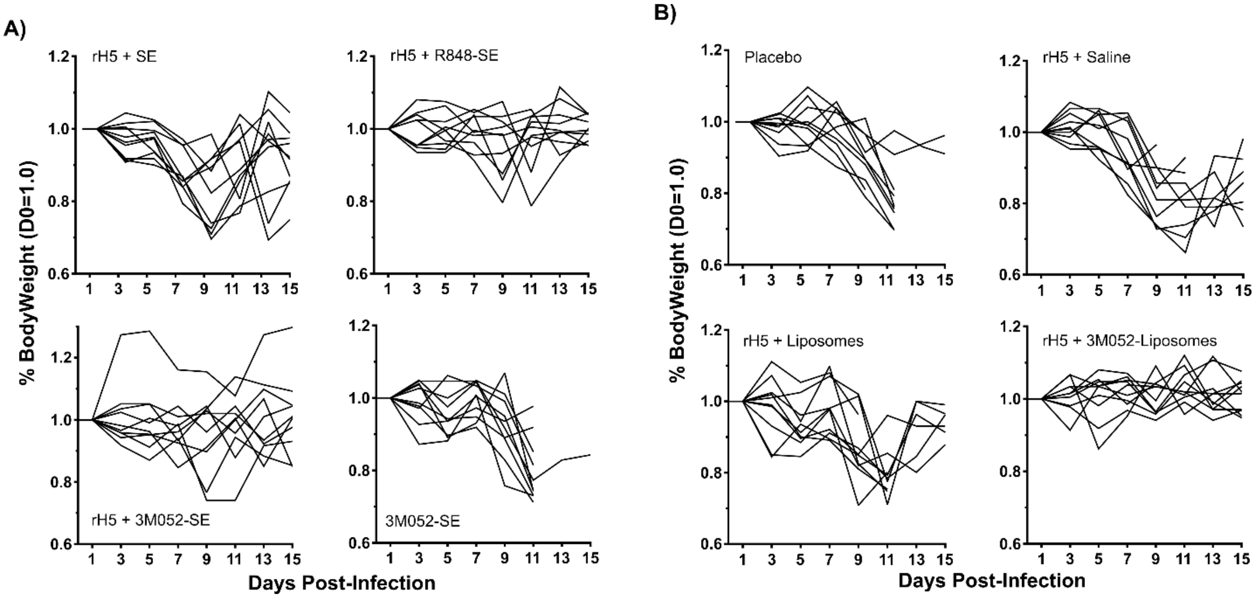


**Supplementary Figure S2: Weight loss observed in mice immunized with a recombinant H5N1 antigen in combination with 3M-052 adjuvant formulations.**

Mice (n=10/group) were immunized once with a recombinant HA protein (rHA, A/VN/1203/04, Protein Sciences Inc.) and challenged 21 days later with $10^6$ PFU of A/VN/1203/04 via the intranasal route. Relative body weights for each mouse were determined daily post-challenge for up to 15 days, or until animals lost 25% of their day-0 starting weight. For both SE (A) and liposomal (B) emulsion, inclusion of 3M-052 reduced weight loss following challenge, with animals receiving R848-SE also showing reduced weight loss. These results are consistent with survival findings.

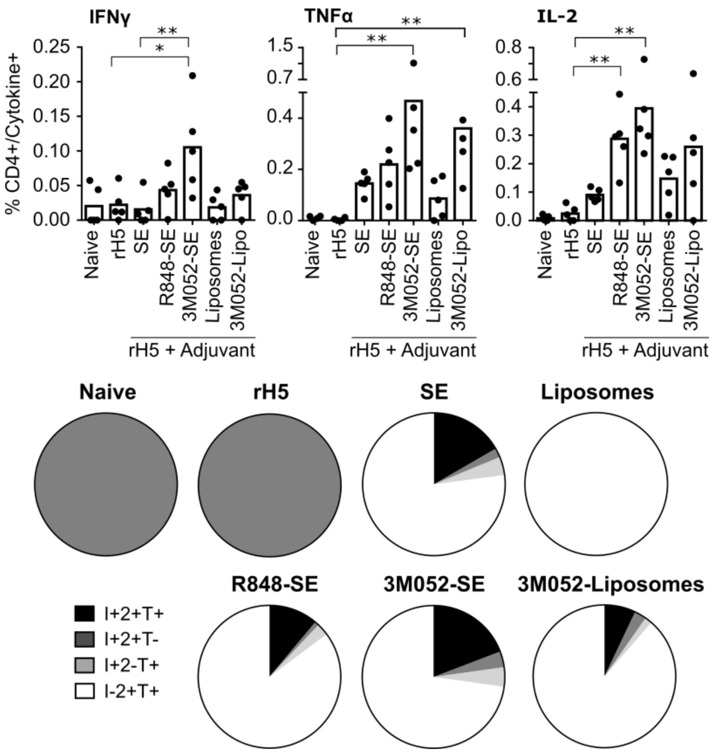


**Supplementary Figure S3: CD4 T-Cell Responses in Mice Immunized with Formulated 3M052 Adjuvants.**

Animals were immunized once with rHA protein (A/VN/1203/04) in combination with adjuvants as indicated. Seven days post immunization, splenocytes from euthanized mice (n=5/group) were analyzed for cytokine stimulation following stimulation with rHA. Cytokine secretion patters for IFNγ (I), TNFα (T), and IL-2 (2) were determined to investigate the induction of a Th1 CD4+ T-cell response. The relative percentage of polyfunctional T-cells was also determined. Significance between groups was determined by one-way ANOVA (*$p<0.05$, **$p<0.005$).

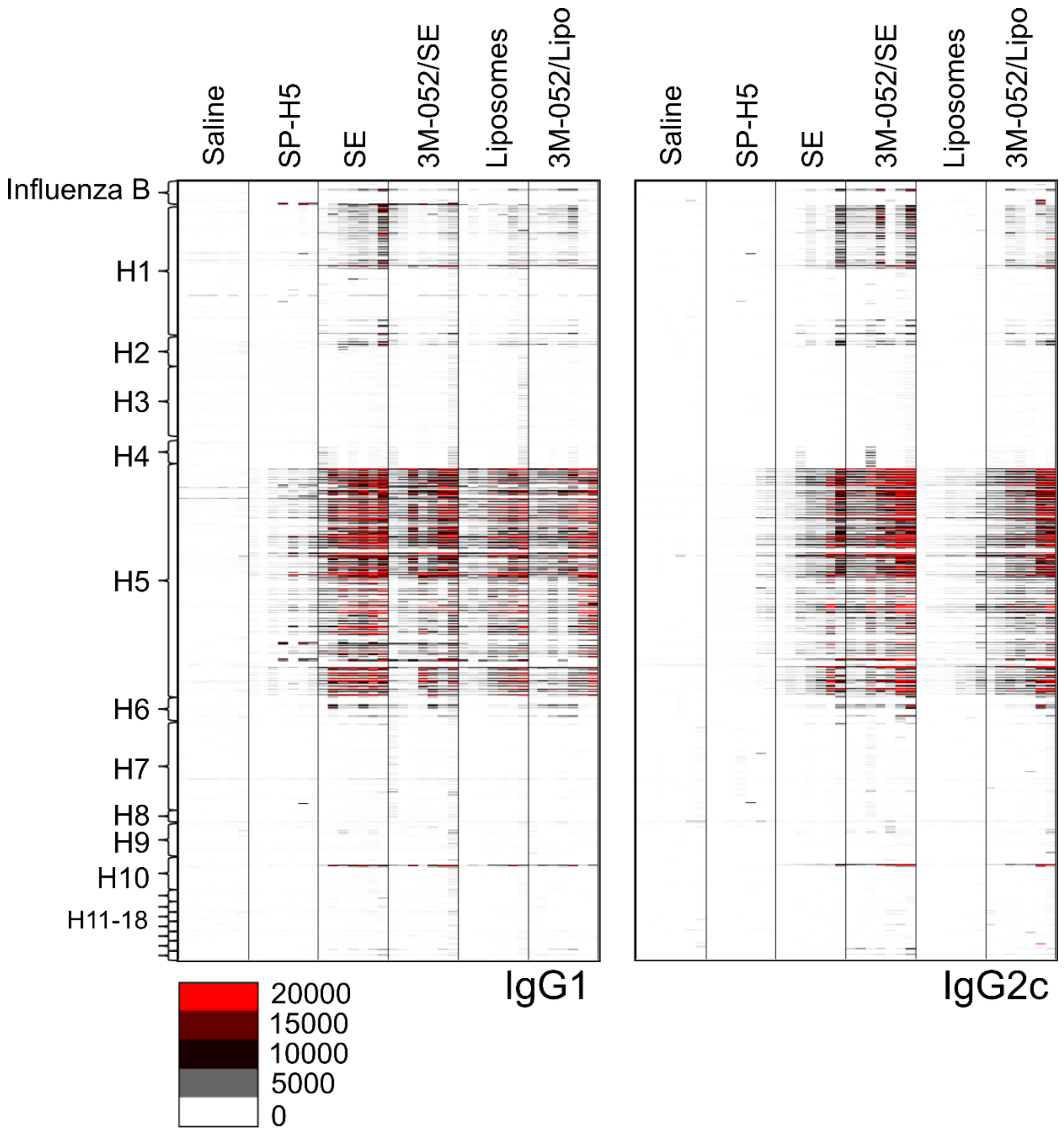


**Supplementary Figure S4: Heat map showing the median fluorescence intensity and breadth of antibodies induced by 3M-052 adjuvant formulations to different HA molecules in a high density HA array.**

HA proteins, sorted by virus subtypes are grouped into rows, with samples from individual mice (n=7/group) presented in columns. Signal intensities, defined as median fluorescence for both IgG1 (532nm) and IgG2c (647nm) are shown. Red indicates strong positive reactivity; black, intermediate reactivity; white, no reactivity.

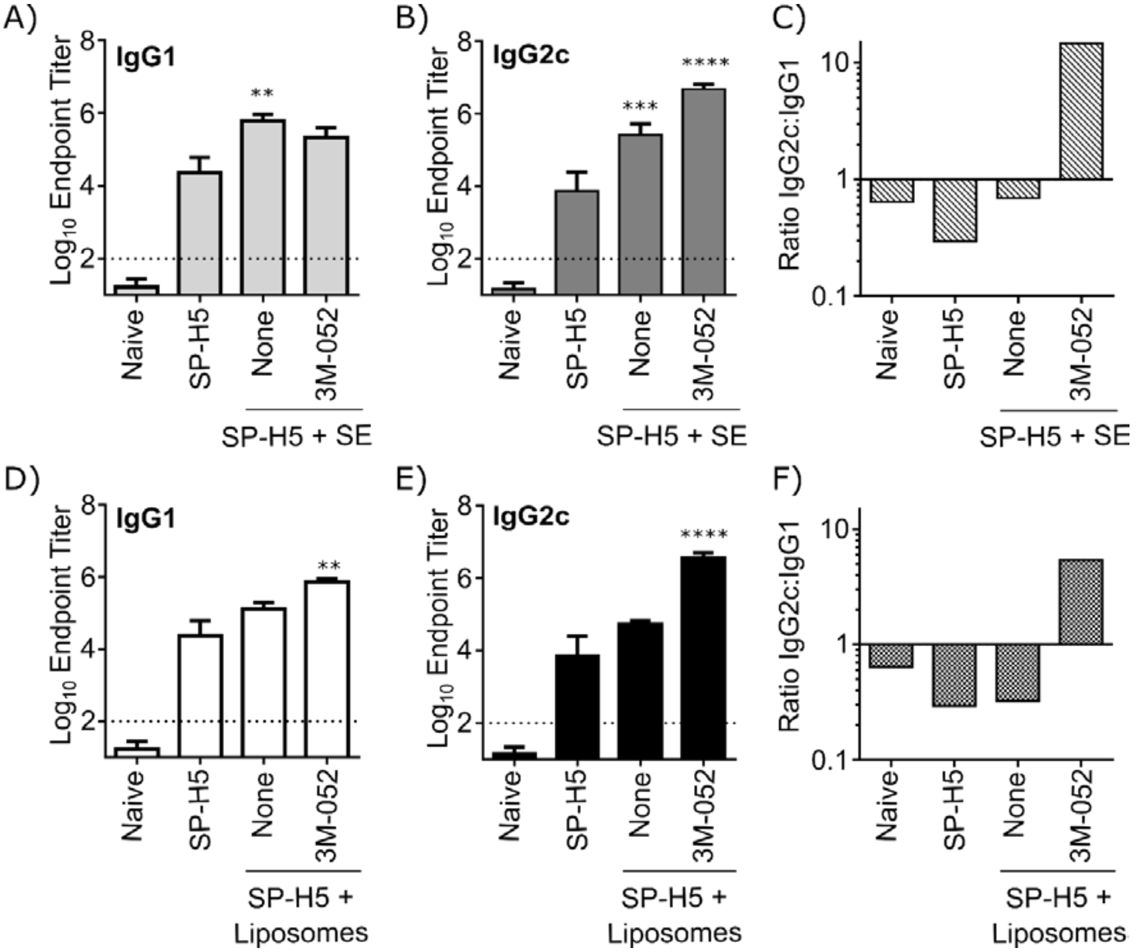


**Supplementary Figure S5: ELISA Titers in Serum from mice immunized with 3M-052 Adjuvants.**

Mice (n=5/group) were immunized twice with a split VN1203 H5N1 antigen in combination with 3M-052 adjuvant formulations. Thirty-five days following a boost immunization, serum was collected from all animals. VN1203 HA specific antibody titers were analyzed by ELISA. Addition of 3M-052 to either emulsion (A-C) of liposomal (D-F) resulted in a specific and significant increase in IgG2c titers, consistent with finding obtained on the HA protein array. Significance is relative to titers observed with antigen alone, and was determined by One-way ANOVA (**$p<0.005$, ***$p<0.0005$, ****$p<0.0001$).

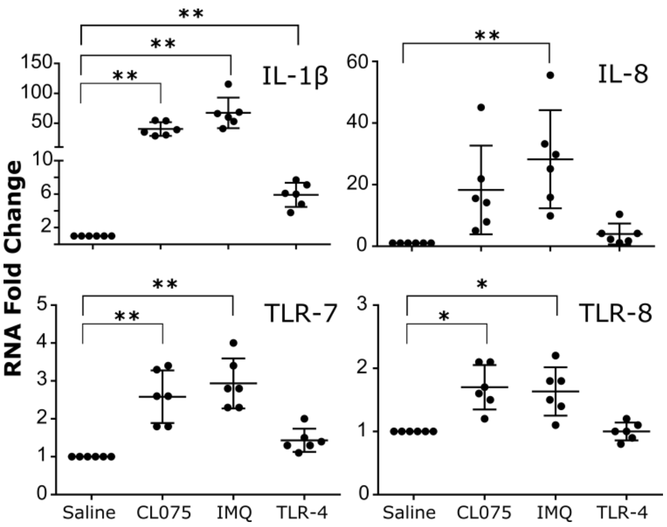


**Supplementary Figure S6: Stimulation of Ferret Whole Blood with TLR Agonist Compounds.**

Blood from male Fitch ferrets was stimulated for 3 hours with TLR agonists including 100µM CL075 (TLR-7/8), 100µM Imiquimod (IMQ, TLR-7), or 10µM glucopyranosyl lipid A (GLA,TLR-4). Following 24h incubation, mRNA was collected from all samples, and analyzed for changes in expression level by real-time PCR. Changes in mRNA copy number are presented as fold change relative to Saline stimulated controls. Incubation of blood with IMQ induced an increase in TLR-7 and TLR-8 mRNA, as well as significant increases in mRNA for IL-1B and IL-8. This indicates that these ligands are capable of stimulating ferret TLR-7 and 8. Significance was determined by one-way ANOVE (* $p<0.05$, ** $p<0.005$).

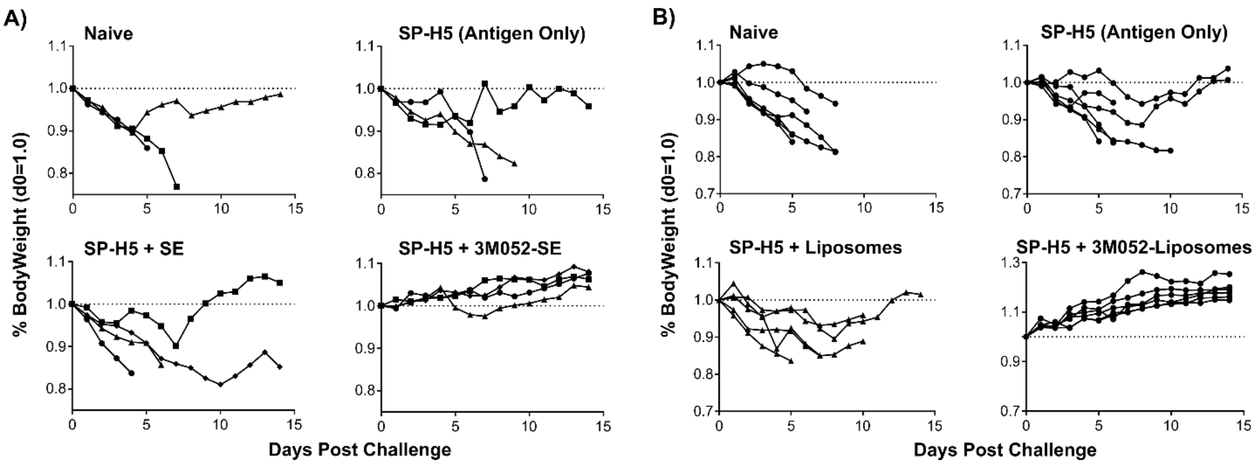


**Supplementary Figure S7: Weight loss observed in ferrets immunized with a split H5N1 antigen in combination with 3M-052 adjuvant formulations.**

Male Fitch ferrets (n=3-4/group) were immunized once with a split HA protein (SP-H5, A/VN/1203/04, Sanofi Pasteur.) and challenged 21 days later with $10^6$ PFU of A/VN/1203/04 via the intranasal route. Relative body weights for each ferret were determined daily post-challenge for up to 15 days, or until animals lost 25% of their day-0 starting weight. For both SE (A) and liposomal (B) emulsion, inclusion of 3M-052 reduced weight loss following challenge.
